# The role of *Mycobacterium tuberculosis* exosomal miRNAs in host pathogen cross-talk as diagnostic and therapeutic biomarkers

**DOI:** 10.3389/fmicb.2024.1441781

**Published:** 2024-08-08

**Authors:** Farwa Mukhtar, Antonio Guarnieri, Natasha Brancazio, Marilina Falcone, Maria Di Naro, Muhammad Azeem, Muhammad Zubair, Daria Nicolosi, Roberto Di Marco, Giulio Petronio Petronio

**Affiliations:** ^1^Department of Medicina e Scienze della Salute “V. Tiberio”, Università degli Studi del Molise, Campobasso, Italy; ^2^Department of Drug and Health Sciences, Università degli Studi di Catania, Catania, Italy; ^3^Department of Precision Medicine in the Medical, Surgical and Critical Care Area (Me.Pre.C.C.), University of Palermo, Palermo, Italy; ^4^Department of Bioinformatics and Biotechnology, Government College University, Faisalabad, Pakistan

**Keywords:** microRNAs, tuberculosis, diagnostics, therapeutics biomarkers, host pathogen interactions, immune response, bacterial exosomes, intercellular pathogen

## Abstract

Tuberculosis (TB) is a global threat, affecting one-quarter of the world's population. The World Health Organization (WHO) reports that 6 million people die annually due to chronic illnesses, a statistic that includes TB-related deaths. This high mortality is attributed to factors such as the emergence of drug-resistant strains and the exceptional survival mechanisms of *Mycobacterium tuberculosis* (MTB). Recently, microRNAs (miRNAs) have garnered attention for their crucial role in TB pathogenesis, surpassing typical small RNAs (sRNA) in their ability to alter the host's immune response. For instance, miR-155, miR-125b, and miR-29a have been identified as key players in the immune response to MTB, particularly in modulating macrophages, T cells, and cytokine production. While sRNAs are restricted to within cells, exo-miRNAs are secreted from MTB-infected macrophages. These exo-miRNAs modify the function of surrounding cells to favor the bacterium, perpetuating the infection cycle. Another significant aspect is that the expression of these miRNAs affects specific genes and pathways involved in immune functions, suggesting their potential use in diagnosing TB and as therapeutic targets. This review compiles existing information on the immunomodulatory function of exosomal miRNAs from MTB, particularly focusing on disease progression and the scientific potential of this approach compared to existing diagnostic techniques. Thus, the aim of the study is to understand the role of exosomal miRNAs in TB and to explore their potential for developing novel diagnostic and therapeutic methods.

## Introduction

Tuberculosis (TB) is a severe bacterial infectious disease that poses a significant threat to global health and sanitation (Paul, 2024). In 2022, 10.6 million individuals worldwide were afflicted with TB. The disease can affect people of any age or gender, with an estimated 5.8 million cases among adult men, 3.5 million cases among adult women, and 1.3 million cases among children. These figures highlight the significant impact of TB on the global population. Additionally, an estimated 1.30 million fatalities from TB were reported in 2022. This figure is nearly back to 2019 levels, down from the peak estimates of 1.4 million in both 2020 and 2021 (World Health Organization, 2023).

Six million cases of TB were reported globally in 2021 (Bagcchi, 2023). However, it was observed in 2020 that TB has been steadily rising, especially among adolescents between the ages of 10 and 24 (Snow et al., 2020). TB is a highly transmissible respiratory illness caused by *Mycobacterium tuberculosis* (MTB) particles that infected individuals disseminate into the environment. Nevertheless, MTB cannot infect individuals unless their immune system is compromised (Moule and Cirillo, 2020).

MTB can infect nearly any part of the human body but primarily targets the lungs. It is important to distinguish between TB disease and MTB infection, as not all infections result in active TB (Karpinski, 2024). Furthermore, individuals with latent TB infection (LTBI) can be infected and show no symptoms, even though they typically test positive for TB through skin or blood tests. Proper TB tests are designed to detect latent infections, so a negative test result generally indicates the absence of infection? (Carranza et al., 2020).

Moreover, LTBI patients are among the few who can develop active TB infection (ATBI) at some point in their lives and play a significant role in spreading the disease (Ferluga et al., 2020).

MTB can persist by evading the host's immune system, mainly by altering its immune cells and the host's Micro RNA (miRNAs). These strains, such as MTB, reside in host macrophages, making them challenging to identify and eliminate (Bo et al., 2023). After transcription, miRNAs are crucial in regulating the expression of potential genes. They can bind to the 3′ untranslated regions of the messenger RNA (mRNA), causing either mRNA degradation or suppressing the translation process (Negrini et al., 2022).

The process of gene regulation by miRNAs is well-known and extensively discussed. Scholars have further revealed interactions between miRNAs and the immune system. Moreover, miRNAs are involved in the development of immune cells and influence their effectiveness, including macrophages, T cells, and B cells. Notably, three particular miRNAs—miR-155, miR-125b, and miR-21—have been proven to play roles in different immune responses to MTB (Zhao et al., 2019).

These miRNAs are critical in preserving fundamental signaling pathways and immune responses, significantly helping to determine the progression of the infection. They modulate the production of different inflammatory cytokines and play a significant role in regulating the activation and functions of macrophages, which are crucial for the clearance of TB and MTB (Singh et al., 2021).

Certain factors in the emergence of drug-resistant (DR) and multiple drug-resistant (MDR) TB complicate the diagnosis and treatment of TB. Therefore, developing new biomarkers with exceptional sensitivity and specificity is crucial for diagnosing TB (Jumat et al., 2023). Traditional approaches such as smears and MTB culture have limitations in determining the cause of TB. Some exosomal miRNAs are involved in TB development and can be distinguished from small RNAs that do not affect the host's immune response (Sharma et al., 2023).

For scientists studying the mechanisms of living organisms or the impact of different medications, it is equally attractive to investigate the actions of exosomal miRNAs released by MTB on macrophages. This manipulation allows the pathogen to counteract or evade the immune system, thereby maintaining its infection. Such miRNAs target genes and pathways linked to immune response processes and may be used for TB diagnosis and potential therapeutic target discovery (Carranza et al., 2021). Research should focus on identifying promising exosomal miRNA targets and developing innovative treatments for TB, highlighting their importance in managing the disease.

In this review article, the authors analyze the latest studies focused on the immunomodulatory properties of MTB exosomal miRNAs. They discuss how these miRNAs influence the host immune response and highlight their potential utility as biomarkers for tracking the progression and severity of MTB infection. The review provides an overview of the molecular mechanisms by which MTB exosomal miRNAs modulate immune functions and considers their implications for diagnostic and therapeutic strategies in TB management.

## Understanding exosomes and miRNAs: formation and composition

**Figure 1:**
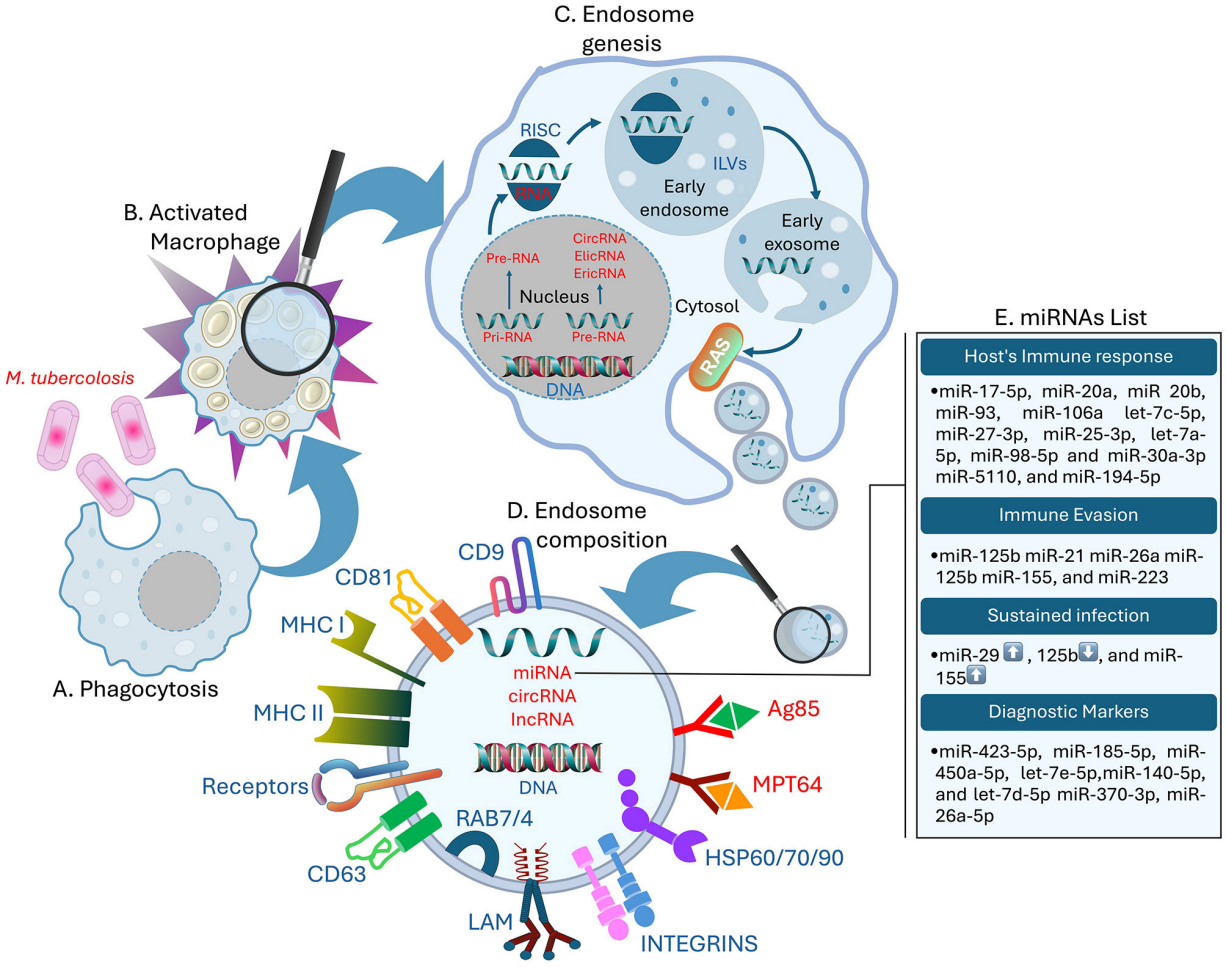
Role of exosomal miRNAs in the host's immune response. **(A)** MTB infecting the host macrophages, **(B)** activated Macrophage, **(C)** formation of the Drosha complex, ILVs, and release of exosomes from the host macrophages with bacterial non-coding RNAs (RNAs in red), **(D)** structure of the MTB exosome containing the necessary proteins. LAM, CD cluster, MHC-I/II, Receptors like PRRs, T-cell receptors, and Scavenger receptors; bacterial antigens like Antigen 85 and non-coding RNAs (RNAs in red), **(E)** list of exosomal miRNAs involved in host's immune response regulation, immune evasion, and up ↑ and down ↓ expression of sustained infection miRNA, and diagnostic markers.

Exosomes play a significant role in mediating cell-to-cell communication and are involved in the pathogenesis of MTB. These small membrane vesicles range in size from 30 to 150 nm in diameter and can be released into the extracellular matrix by virtually any type of cell (Dreyer and Baur, 2016; Li et al., 2020; Kang et al., 2021). Exosomes originate through endocytosis, forming small cup-shaped organelles known as early endosomes. These early endosomes facilitate the encapsulation of extracellular proteins, other molecules, and specific cell membrane receptors (Dreyer and Baur, 2016; Kalluri and LeBleu, 2020). During the transition from early endosomal to late endosomal identities, cargo molecules are concentrated on the early endosome's surface. Subsequent budding processes transport these cargo molecules into the intraluminal vesicles (ILVs) (Figure 1). These processes can be further organized into Endosomal Sorting Complex Required for Transport (ESCRT)-dependent and ESCRT-independent pathways, depending on the presence of ESCRTs in a cell (Eitan et al., 2016; Rayamajhi and Aryal, 2020; Gurunathan et al., 2021).

There are two primary biosynthetic miRNA pathways: the canonic and non-canonic signaling pathways (Sun et al., 2018). According to Hill and Tran, the canonic pathway is the principal route for miRNA biogenesis (Hill and Tran, 2022). RNA polymerase II transcription in the nucleus generates long primary miRNA with hairpin structures called pri-miRNA (Yu et al., 2016). Subsequently, the Drosha complex, which includes Drosha, Ribonuclease III (RNase III), double-stranded RNA–binding protein, DiGeorge syndrome critical region 8, and other partner proteins, cleaves the prior-miRNA into pre-miRNA with a stem-loop structure. In this stage, the pre-miRNA is processed to be transported in the cytoplasm. Once in the cytoplasm, the RNase III endonuclease Dicer processes the hairpin duplex into double-stranded miRNAs, producing a mature miRNA, the complementary strand, and the Argonaute proteins (Ago proteins), which are partial small interfering RNA (siRNA)-like molecules (Matsuyama and Suzuki, 2019; Kilikevicius et al., 2022). One of the two chains is chosen as the endogenous miRNA, whereas the second chain is generally cleaved (Riahi Rad et al., 2021) (Figure 1).

Following the MTB invasion of the respiratory organs, the immune cells, such as macrophages and dendritic cells, engulf the pathogen (Sia and Rengarajan, 2019). Macrophages and dendritic cells of the innate immune system detect MTB pathogen-associated molecular patterns (PAMP) through damage-associated molecular patterns (DAMP) or with the help of membrane surface pattern recognition receptors (PRRs) (Boom et al., 2021). Alveolar macrophages (MS) are particularly targeted in the initial stage of MTB invasion (Cohen et al., 2018). Recently, more research has focused on various types of receptors, such as scavenger receptors (Linares-Alcántara and Mendlovic, 2022), in connection with phagocytosis (Choudhuri et al., 2020). Therefore, to transport MTB within the cytoplasm and to form and translocate it into the phagosomes, immune cells must synthesize sphingomyelin on the cell surface (Niekamp et al., 2021). The phagosome pH drops during endosome-phagosome fusion, and then it fuses with lysosomes to create acidified phagolysosomes, which are essential for MTB suppression or elimination (Rai et al., 2022). This process, known as phagocytosis, involves LC3-associated phagocytosis (LAP) (Weiss and Schaible, 2015). After TB infection, macrophages activate bioactive 1,25-dihydroxy vitamin D (1,25D) via the vitamin D receptor, enhancing antimicrobial peptide synthesis (Cathelicidin Antimicrobial Peptide and β-defensin 2) and inflammatory proteins (IL-1β and IL-8), contributing to immunomodulation. MTB evades immune responses through strategies like apoptosis and modulation of innate immune cell responses (Wang et al., 2019). Multi Vesicular Bodies (MVBs) can fuse with lysosomes and, when subjected to lysosomal acid and proteolysis, become degraded. Moreover, MVBs can fuse with the plasma membrane and release the ILVs into the external milieu. Exosomes can also be formed by budding directly through the cytoplasmic membrane, assupported by previous studies (Eitan et al., 2016; Kalluri and LeBleu, 2020). When exosome secretion is inhibited, there is an increase of MVBs enhanced by lysosomal degradation (Eitan et al., 2016). The release and the fusion of exosomes with recipient cells membrane are closely regulated by the Ras superfamily. Several Rab proteins act as molecular signals in the movement of MVBs from one compartment to another. These proteins play a notable role in regulating vesicle transport, as highlighted in recent studies (Rayamajhi and Aryal, 2020; Gurunathan et al., 2021). Furthermore, the release of exosomes is facilitated by Ral A and B Guanosine Triphosphatases (RalA/B GTPases), which regulate effectors and lipid metabolism. These GTPases also control phospholipases D1 and D2, which are involved in maintaining MVB equilibrium and exosome cargo formation (Zago et al., 2019; Ghoroghi et al., 2021). The process described by Wickner & Rizo elucidates how Rab GTPase facilitates the assembly of membrane-bound soluble N-ethylmaleimide-sensitive factor attachment protein receptors into tetrameric coiled-coil complexes at both exosomal and receptor cell membranes (Wickner and Rizo, 2017), mediated by tethering proteins that colocalize the two membranes to allow them to come close (Borchers et al., 2021). In addition, exosome proteins include Cluster of Differentiation (CD) 63, CD81, CD9, flotillin, Alix, and Tumor Suceptibility Gene 101 (TSG101) (Figure 1). These proteins are implicated in the creation of exosomes, as noted by Gurunathan et al. (2021) in 2021. The exosomes biogenesis includes several pathways and electiveprocesses that determine their structural, transfer route, and cargo diversity acquired by cells from different sources. Overall, exosomes are crucial in cellular communication due to their ability to encapsulate diverse molecules through well-defined pathways. Their formation and release are complex processes regulated by various proteins and pathways, emphasizing their diverse roles in cellular communication and transport. Thus, their involvement in the immune response to MTB involves complex interactions between different cells and molecules, highlighting the pathogen's ability to evade immune defenses.

### The function of exosomes in MTB-infected hosts

Microvesicles contain diverse cargo, such as nucleic acids (e.g., miRNA, lncRNA, mRNA, DNA), proteins, lipids, and metabolites (Kugeratski et al., 2021). Exosomes, a subset of microvesicles, play a significant role in intercellular and intracellular signaling and communication between cells to regulate cellular processes and immunological defenses.

MTB relies on the Specialized Secretion System A2 (SecA2) and ESAT-6 Secretion System 1 (ESX-1) secretory systems for cell membrane degradation (Cheng and Schorey, 2019). Previous research by Tiwari et al. demonstrated the crucial role of exosomes in transferring genetic material, proteins, and other molecules within and between cells.

PRRs recognize these exosomes as PAMPs of the LAP pathway, triggering inflammasome activation (Tiwari et al., 2019). The cationic liposome driven by MTB-infected Mesenchymal Stem Cells (MSCs) compels macrophages to release pro-inflammatory cytokines, which include Tumor Necrosis Factor-alpha (TNF-α), C-C Motif Chemokine Ligand 5 (CCL5), and Inducible Nitric Oxide Synthase (iNOS). These factors escalate Toll-Like Receptor 2 and Toll-Like Receptor 4 (TLR2/4) and Myeloid Differentiation Primary Response 88 (MyD88) to induce inflammation and immune response (Liu et al., 2021). Consequently, the macrophage exosomes released by MTB-infected cells play a significant role in monocyte differentiation. This process involves the activation of specific molecules and proper pathways, resulting in the generation of functional macrophages (Singh et al., 2023). These exosomes are released following stimuli by lipopolysaccharide (LPS) and interferon-gamma (IFN-γ), most frequently by macrophages. These vesicles can bind soluble endoplasmic reticulum aminopeptidase-1; thus, this study was intended to address this interaction and the effects it has on macrophages' phagocytosis and nitric oxide production (Goto et al., 2018).

Additionally, macrophages can engulf necroptotic exosomes, leading to the secretion of pro-inflammatory cytokines such as TNF-α, IL-6, and C-C Motif Chemokine Ligand 2 (CCL2); Chemokine (Shlomovitz et al., 2021). Exosomes secreted by Antigen-Presenting Cells (APCs) contain Major Histocompatibility Complex class I and class II (MHC-I/II), which present antigenic information to T lymphocytes, thereby triggering specific immune reactions (André et al., 2004; Ramachandra et al., 2010). Active T cells promote the production of exosomes from miR155-loaded dendritic cells (DCs), strengthening the activation of appropriate T cells (Okoye et al., 2014). Furthermore, T helper 1 (Th1) receiving let-7b-containing exosomes from Treg cellscounterbalance the excessive inflammatory response (Lindenbergh et al., 2019). Activated T lymphocytes transfer genetic material to DCs, aiding in the innate immune response to curb MTB infection (Torralba et al., 2018), while mitochondrial components play a crucial role in detecting and relocating DAMPs to various biological processes (Koenig and Buskiewicz-Koenig, 2022). Moreover, exosomes induce tumor-associated macrophage-derived Cell Line 1 (THP-1) autophagy (Sun et al., 2021b), and macrophage stimulation by exosomes secreted from MTB-infected neutrophils has also been observed (Alvarez-Jiménez et al., 2018). This stimulation provokes the formation of reactive oxygen species (ROS) and the generation of autophagy to eradicate MTB infection.

During infection, infected macrophages shed exosomes that deliver miR-18a into target cells; this specific miRNA interferes with autophagy, thereby promoting the resilience of MTB within the macrophages. A 2020 study conducted by Yuan et al. (2020) demonstrated the regulation of the Ataxia Telangiectasia Mutated-AMP-activated Protein Kinase (ATM-AMPK) autophagic pathway has been achieved. Macrophage-derived exosomes can also suppress the T-cell receptor expressed on CD4 + T cells and IL-2 production, as pointed out by Athman et al. in 2017 (Athman et al., 2017). Singh et al. (2011) found a reduction in IFN-γ levels with kinetics comparable to the reduction of CD64 or MHC-II expression in infected macrophages. Considering these findings, exosomes play a critical role in cellular communication and immune regulation, particularly in the context of MTB infection. They enhance immune responses and contribute to disease progression through various molecular pathways and interactions, making them potential targets for TB diagnosis and therapy.

## Exosomal miRNAs: potential and synthesis

miRNAs are naturally occurring small RNA molecules of around 18–24 nucleotides that do not code for protein andare preserved throughout various evolutionary states (Iacomino, 2023). Moreover, miRNAs are pivotalin the modulation of several important biological processes, involving cell proliferation, differentiation, migration, apoptosis, and autophagy. They achieve this bytargeting gene mRNAs at the 3′-untranslated region (Song et al., 2019; Farina et al., 2020; Riahi Rad et al., 2021; Zhu et al., 2021).

miRNAs can coordinate multiple biological activities due to their complex regulation mechanisms. Their ability to remain constant throughout evolutionary stages emphasizes their evolutionary significance and adaptability in controlling gene expression. MiRNAs bind to target gene mRNAs at specific 3′-untranslated regions to alter genetic networks with remarkable precision. The complex biosynthesis of miRNAs involves transcription, cleavage by Drosha and Dicer, and the selection of functional miRNA strands, ensuring effective Ago protein-mediated post-transcriptional gene silencing.

### The functions of exosomal miRNAs in the host

Exosomal miRNA from MTB-infected macrophages can be isolated using centrifugation techniques, making them suitable biomarkers for MTB infection. Zhang et al. (2019) found that exosomes from MTB-infected macrophages contain miR-20b-5p, while non-infected macrophage exosomes did not have this specific microRNA (Zhang et al., 2019). Recently, the same authors employed high-throughput sequencing to discover miRNAs in exosomes secreted by macrophages infected with *Mycobacterium bovis*.

The research revealed that the expression of 20 exosomal miRNAs unfolded increased in the samples of infected patients, while seven exosomal miRNAs decreased in the infected group compared to the non-infected group. Specifically, higher levels of let-7c-5p, miR-27-3p, miR-25-3p, let-7a-5p, miR-98-5p, and miR-30a-3p, and lower levels of miR-5110 and miR-194-5p were observed in the infection group (Zhan et al., 2022). In a 2016 study, Kumar et al. compared the quantitative alterations of several exosomal miRNAs in infected macrophages and the lung, spleen, and lymph nodes of MTB-infected mice to those in a control group. During MTB infection, miR-17-5p was found to be downregulated in both macrophages and mice. Other miRNAs from the same family, including miR-20a, miR-20b, miR-93, and miR-106a, were also downregulated in infected macrophages. The study revealed that miR-17 regulates the levels of Myeloid cell leukemia sequence 1 (Mcl-1) and its transcriptional activator Signal Transducer and Activator of Transcription 3 STAT3 in the context of MTB infection. By targeting Mcl-1 and STAT3, miR-17 plays a role in regulating autophagy. This finding highlights the importance of miRNAs, specifically miR-17-5p, in modulating autophagy and host responses during MTB infection (Kumar et al., 2016).

Exosomal miRNAs might be valuable for differentiating TB from other lung disorders. Wang et al. investigated the differences in exosomal miRNA in pulmonary adenocarcinoma (ADC), TB, and other diseases by evaluating their pleural distribution using qPCR. They found miR-205-5p, miR-429, miR-483-5p, miR-375, miR-200b-3p, and miR-200c-3p to be high in adenocarcinoma exosomes compared to tubercular and other pathology, while a downregulation of specific miRNAs, such as miR-3614-5p and miR-150-5p, was observed in malignant pleural. In addition, exosomes from TB were found to be laden with 148a-3p and 150-5p, which are lower compared to exosomes from non-cancerous tissues, showing contrasting results regarding the expression levels of miR-451a (Wang et al., 2017). Exosome expression from LTBI, active tuberculosis infection (ATBI), and ADC patients' blood was evaluated by small RNA (sRNA) sequencing (Guio et al., 2022). Levels of miR-210-3p and miR-143-3p in serum exosomes from patients with LTBI decreased, while miR-20a-5p was upregulated in exosomes from patients' serum. Regarding ATBI serum exosomes, miR-23b, miR-17, and miR-181b-5p were downregulated, while miR-584 was upregulated. Finally, for ADC patients, 15 downregulated miRNAs were found, including miR-320a, miR-185-5p, miR-144-3p, let-7f-5p, and miR-199b-3p (Guio et al., 2022).

Exosomal miRNAs isolated from MTB-infected macrophages exhibit distinct profiles that hold promise as diagnostic biomarkers for TB. In light of this evidence, the differential expression of miRNAs in various clinical contexts underscores their potential utility in distinguishing TB from other lung disorders and monitoring disease progression. These findings advocate for further research into exosomal miRNAs to refine their diagnostic accuracy and clinical applicability in TB management.

### miRNA-mediated manipulation by MTB

An evaluation score of exosomal miRNA found in MTB patients was determined, revealing their promise for rapid and non-invasive TB diagnosis. Research by Kaushik et al. (2021) showed that plasma exosomal miR-185-5p expression was significantly upregulated in the TB patient group compared to the healthy controls. Other authors also indicated that integrating miR-185-5p with other markers could significantly improve the diagnosis of TB.

A study conducted by Tu et al. (2019) identified higher levels of exosomal miR-423-5p in the plasma of TB patients. The TB diagnostic model achieved a score of 0. 908 with a 10-fold cross-validation mean prediction accuracy of 78%. The model demonstrated an accuracy of 18% and could differentiate between ATBI patients and healthy subjects, reflecting the findings of Kaushik et al. (2021). In the smear exosomes of LTBI patients, the levels of miR-450a-5p, let-7e-5p, miR-140-5p, and let-7d-5p were high. Additionally, in patients with LTBI, miR-370-3p and miR-26a-5p levels were higher than those in ATBI patients (Lyu et al., 2019). The results showed an escalating trend in the levels of these miRNAs among Healthy Control (HCs), LTB, and ATB patients. This distribution can be helpful in the recognition of different states of MTB infection (Lyu et al., 2019).

Alipoor et al. also found that the concentration of specific miRNAs in serum exosomes was significantly higher in patients with TB compared to healthy individuals. When these miRNAs were studied in combination with sputum smears, the diagnostic sensitivity for TB improved (Alipoor et al., 2019).

These research findings indicate that specific exosomal miRNAs hold significant potential for the rapid and non-invasive diagnosis of TB, demonstrating high diagnostic accuracy and the ability to differentiate between various stages of MTB infection. These findings highlight the importance of miRNA markers in improving TB diagnostics when combined with other methods, such as sputum smears, plasma analysis, and cross-validation models.

### Role of exosomal miRNAs in immune regulation

The intrinsic properties of MTB enable it to survive through specific mechanisms. Following MTB infection, post-transcriptional regulation via miRNAs modulates target genes and the associated biological and immune processes. Indeed, miRNAs can inhibit translation or lead to mRNA degradation by specific mRNA target sites. This finding stems from the transcription mechanism of miRNA genes through RNA polymerase II and their post-transcriptional processing via endonucleases (Singh et al., 2022). miRNAs exhibit immunomodulatory functions for numerous immune cells, such as macrophages, T cells, and dendritic cells, playing a critical role in resistance against TB. This factor is significantly involved in the immune response to MTB in affected tissues. Ruiz-Tagle et al. (2020) provided experimental evidence on how the pathogen affects host cellular signaling through miRNAby analyzing dendritic maturation and its reference to the activation of T cells. The results confirm that miR-29 can decrease IFN-γ levels and change the T-cell response. Therefore, while IFN-γ is downregulated, T cells are actively triggered, and macrophages are reactivated. Through this manipulation, pathogens can remain dormant when the immune system is closing in and simultaneously reproduce (Ruiz-Tagle et al., 2020). Various miRNAs participate in the regulation of the immune response in MTB infection, managing the activities of macrophages, dendritic cells, and T cells. For instance, miR-125b, miR-155, and miR-223 significantly affect cytokines, cell cycle, and cell death, all related to the immune system (Yang et al., 2021).

Research conducted by Kozlov et al. (2023) has demonstrated that MiR-29 can influence or regulate IFN-γ, indicating that miRNAs play a role in the regulation of immune responses. Sarkar et al. showed that the downregulation of miR-125b can counteract the upregulation of TNF-α. Although TNF-α is an inflammatory agent beneficial for immune responses, excessive levels can inhibit immune reactions. MiR-125b targets the NF-κB pathway, which is significant for macrophages as it helps prevent prolonged inflammation, thereby aiding the eradication of MTB (Sarkar et al., 2024). In a recent study, Sun et al. explored the differences in infection levels when TNF-α is inhibited by miR-125b. This inhibition may occur when macrophages lose their ability to control and regulate the infection. These studies focus on understanding how miRNAs regulate host defense signaling pathways by targeting specific genes (Sun et al., 2021a).

Thus, the regulatory role of miRNAs, such as miR-29 and miR-125b, in modulating immune responses and host cellular signaling highlights their critical involvement in MTB survival and immune evasion, making them significant targets for understanding and potentially controlling TB infection. It is compelling to discuss the various processes by which MTB manipulates cellular elements. Among these processes, the regulation of miRNAs is notable, as MTB utilizes them to persist and proliferate. Understanding the pathogen's virulence involves examining how the host's miRNA expression, immune status, and cellular environment are affected.

## Key miRNAs and their roles

According to a study by Alijani et al., there is a direct correlation between elevated levels of miR-155 and macrophage differentiation and functionality. This demonstrates how the bacteria might affect the immunity of the host. MTB can downregulate elements that promote anti-inflammatory processes, which are advantageous during an ongoing infection, by upregulating miR-155 (Alijani et al., 2023). Kulshrestha et al. discovered that miR-21 helps the bacteria grow by neutralizing genes that would otherwise cause the infected cells to die. Furthermore, MTB inhibits the miR-125b gene, which is essential for controlling the TNF-α cytokine, a major component of the body's fight against TB. Reduced TNF-α production results from lower levels of miR-125b, which lowers the TNF-α production, reducing the host's ability to combat the infection (Kulshrestha et al., 2019). Fu et al. (2020) highlighted the pathogen's ability to alter host immune responses for its survival by discussing how this manipulation of miRNAs, namely the inhibition of miR-125b, promotes the vulnerability of the host to MTB.

Kundu and Basu established that miR-26a plays a significant role in the immune response concerning MTB infection; it pertains to regulating cytokines belonging to the pro-inflammatory subdivision. This allows the pathogen to stay within the host because it avoids the aggression of the activated macrophages (Kundu and Basu, 2021).

According to Hu et al., miR-26a modulates a signaling pathway concerning several genes involved in immune response and inflammation mechanisms. Furthermore, miR-132 is linked with target genes that participate in the immunity reaction of the host organism. Discriminating from the cell aspect, it is noted that there is an upregulation of TB in cells, which, in turn, affects the production of other essential cytokines, such as TNF-α and IL-6, which are known to boost immunity. Therefore, this mechanism helps MTB escape elimination by the host's immune system and remains less toxic or capable of evoking an immune response (Hu et al., 2020). These findings are corroborated by Daniel et al. (2022) who noted that the host defense pathways in response to bacterial invasion could be affected by miR-132.

According to Wang et al., miR-29a can influence IFN-γ, hindering the immune system's ability to suppress MTB. Comparative analysis of the target miRNA focused on apoptosis pathways carried out by J. Wang et al. led to the discovery of the crucial role of miR-29a. This creates a cellular structure that enhances the growth of these bacteria. More importantly, IFN-γ mRNA is not the target of miR-29a; thus, the interaction between the two molecules is not the same. This is because IFN-γ plays a role in combating MTB as it stimulates the activation process of macrophages that are instrumental in the immune response (Wang et al., 2018). On the same note, miR-29a has also been observed to regulate the synthesis of another cytokine, IFN-γ. Specifically, miR-29a focuses on proteins implicated in apoptosis regulation, which will gradually pinpoint whether it is beneficial to hold cells alive soon after infection or otherwise eliminate them (Ruiz-Tagle et al., 2020).

Therefore, it is necessary to understand and determine the targets and functions of specific miRNA molecules, including miR-26a, miR-132, and miR-29a. Such understanding yields significant knowledge on how and through which MTB can evade several host cellular processes to increase survival (Crane et al., 2018).

The functions of these miRNAs in immune system regulation and their significance for TB are presented in Table 1. They play crucial roles in immunity, such as inflammation, T cell differentiation, and B cell activation. These processes are essential for the human body's ability to combat infection. These miRNAs have a dual role in modulating host defense and the pathogen's ability to promote infection. Understanding the mechanisms of action and molecular pathways of these miRNAs may provide potential treatment strategies for modulating these miRNAs to disrupt MTB's balance in managing TB.

**Table 1:** miRNAs' roles in immune responses and their impact on TB.

**miRNA**	**Immune system aspect**	**Function**	**Impact on TB response**	**Methodology**	**References**
miR-155	Innate Immunity	Serves as an infection and activation marker, with higher expression in active TB	Indicates active TB, enhances immune response in macrophages	*In vitro*	(Alijani et al., 2023)
miR-125b	Innate Immunity	Modulates inflammatory response by targeting ROCK1	Promotes MTB survival in macrophages, impairing host defense	*In vivo*	(Sun et al., 2021a)
miR-223	Innate Immunity	Regulates myeloid cell function	Balances pathogen clearance with inflammation control, reducing tissue damage	*In vitro*	(Peng et al., 2022)
miR-29	Adaptive Immunity	Modulates inflammatory cytokine production	Overexpression can suppress IFN-γ, leading to inadequate immune response	*In vivo*	(Li et al., 2020)
miR-21	Adaptive Immunity	Modulates T cell differentiation and function	Ensures controlled and effective immune response, avoiding overactivity	*In vitro*	(Zhao et al., 2019)
miR-150	Adaptive Immunity	Involved in the regulation of immune responses, particularly B cell differentiation and antibody production	Influences B cell repertoire, potentially enhancing long-term immunity against MTB.	*In vivo*	(Sinigaglia et al., 2020)

## Diagnostic potential of mirnas in TB

### Challenges in current TB diagnostic methods

Currently, three widely-known TB diagnosis methods are the Mantoux tuberculin skin test, two types of IGRA tests, and sputum culture tests. However, these methods have demerits that cause their ineffectiveness and redundancy. The second important method of targeting the affected populace and identifying TB is the Mantoux tuberculin skin test, in which purified protein derivative (PPD) is inoculated into the skin. This test assists in determining the skin's reaction, in other words, whether a person has been in contact with TB bacteria. However, it cannot distinguish between the same individual's current and previous infections. Thus, tests like the QuantiFERON-TB Gold may further determine the immune response to TB antigens in the body, increasing the certainty level in diagnosing TB disease (Yang and Ge, 2018). It has been noted elsewhere that knowledge of how to manage DR-TB and MDR-TB is critical in eradicating this illness. Research suggests that exosomal miRNAs could be utilized to predict disease progression and diagnose either DR-TB or MDR-TB cases (Carranza et al., 2021).

Carranza et al. aimed to determine the baseline and 12-month post-treatment levels of exosomal miRNA in the serum of MDR-TB patients. Following therapy, they observed a drop in the blood levels of exosomal miR-328-3p, miR-20a-3p, and miR-195-5p. Conversely, let-7e-5p and miR-197-3p showed increased expression after therapy. The research also brought to light notable distinctions between the outcomes of those without cancer and those with cancer. Exosomal miRNAs have shown promise as biomarkers for DR-TB diagnosis and MDR-TB prediction. MDR-TB patients had lower serum levels of miR-197-3p and miR-223-3p than healthy controls, while their serum levels of let-7e-5p were higher. Certain miRNAs found in exosomes made from peripheral serum play a role in assessing the therapeutic impact of MDR-TB patients during phases of prolonged treatment. These results, which are related to variations in the serum of MDR-TB patients and healthy individuals, suggest that circulating miRNA can be utilized to assess TB strains' medication sensitivity or resistance (Carranza et al., 2021).

As highlighted by Barry et al., miRNAs in infected patients assist in differentiating TB from other diseases. The evaluation of exosomal miRNAs reveals their high potential to impact TB-related conditions and behaviors. Consequently, these specific miRNA patterns may help diagnose and classify different types of TB. Additionally, exosomal miRNAs have been implicated in evaluating the effectiveness of anti-TB treatments, underscoring their potential to improve TB management (Barry et al., 2018).

Awareness is essential for TB eradication, as it helps design effective strategies to counteract the disease. However, there is currently insufficient literature on the effects of exosomal miRNAs on TB prognosis. The existing research on the influence of exosomes on TB prognosis is limited. Further research is needed to determine the potential of exosomal miRNAs in diagnosing TB and to expand our understanding of their roles in TB development. Identifying diagnostic and treatment biomarkers through exosomal miRNAs is essential for improving TB management.

### miRNAs as biomarkers for TB diagnosis

Due to their TB specificity, miRNAs can be evaluated as biomarkers for the development of early diagnostic techniques (Olsson, 2024). Implementation of miRNAs for TB diagnosing in clinical can significantly improve treatment outcomes through proper and timely identification (Singh et al., 2021).

Three exosomal miRNAs (miR-484, miR-425, and miR-96) were assessed for their diagnostic utility in TB by Alipoor et al. The area under the curve (AUC) values of 0.72, 0.66, and 0.62 for serum exosomal miR-484, miR-425, and miR-96, respectively, were obtained using receiver operating characteristic (ROC) analysis. Furthermore, in TB-infected subjects, the levels of these three serum exosomal miRNAs were associated with smear TB positivity (Alipoor et al., 2019).

Lyu et al. (2019) compared the serum exosomal miRNA profiles of people with LTBI, ATBI, and healthy controls in a cross-sectional study. Four miRNAs were found to be elevated in the LTBI group: hsa-let-7e-5p, hsa-let-7d-5p, hsa-miR-450a-5p, and hsa-miR-140-5p. Furthermore, patients with ATBI had increased expression of five exosomal miRNAs: hsa-miR-1246, hsa-miR-2110, hsa-miR-370-3P, hsa-miR-28-3p, and hsa-miR-193b-5p. These results suggested that serum exosome miRNA patterns may be used to distinguish between LTBI and ATBI. The diagnostic efficacy of incorporating exosomal miRNA data into TB patients' electronic health records (EHRs) showed improved sensitivity and specificity compared to the tuberculin skin test and interferon-gamma release assays (Lyu et al., 2019).

Hu et al. assessed the mean ± SD of six distinct plasma exosomal microRNA types (miR-20a, miR-20b, miR-26a, miR-106a, miR-191, and miR-486) in TB patients and found notable variations when compared to the control group. Including exosomal miRNAs and EHRs in the diagnostic model showed better diagnostic ability. Treatment success rates for patients with pulmonary TB and TB meningitis were reported to be 97%. Additionally, TB cases involving the organs frequently result in pleural effusion, which affects the pleura (Hu et al., 2020).

Kim et al. attempted to detect exosomal miRNAs across a variety of lung lesions, including lung adenocarcinoma, TB, and non-cancerous lung disorders, to differentiate TB from other lung diseases, such as lung cancer and pneumonia. Three miRNAs—miR-148a-3p, miR-451a, and miR-150-5p—were shown to be elevated in TB lesions compared to benign lung lesions using deep sequencing and qRT-PCR to quantify and compare miRNA expression (Kim et al., 2017).

Nine exosomal miRNAs in pleural effusion (PE) samples were found to be significantly different in the lung adenocarcinoma (LAC) group compared to the other two groups in a distinct study conducted by Wang et al. According to the authors, a few of these miRNAs were miR-205, miR-483, miR-375, miR-200c, miR-429, miR-200b, miR-200a, miR-203, and miR-141 (Wang et al., 2017).

The diagnostic potential of other miRNAs, including miR-146a and miR-125b, has also been examined. Numerous studies have demonstrated the impact of miRNAs on immunological responses and disease susceptibility in TB patients, indicating their potential as biomarkers for determining the presence and stage of the disease (Kundu and Basu, 2021).

Liu et al. established that miRNAs are specific and sensitive indicators for disease diagnosis. The authors can effectively diagnose diseases by using miR-155 and miR-29a, along with other specific miRNAs, as biomarkers (Liu et al., 2022).

According to the mentioned research, while discussing the diagnostic capacity of miRNA for TB, the emphasis should be on the specific types of miRNA. The miRNAs indicated in Table 2, including miR-423-5p, miR-185-5p, miR-26a-5p, miR-450a-5p, let-7e-5p, miR-140-5p, let-7d-5p, and miR-3700-3p, strongly affect the expression of the immunological system and the pathogenicity of TB.

**Table 2:** Some critical miRNAs' expression in TB, used as diagnostic markers.

**miRNA**	**Expression**	**Samples extracted**	**References**
miR-423-5p	High	Plasma of TB patients	(Tu et al., 2019)
miR-185-5p	Upregulated	Plasma exosome of TB patients	(Kaushik et al., 2021)
miR-450a-5p, let-7e-5p, miR-140-5p, and let-7d-5p	High	SM LTBI patient, serum exosomes	(Lyu et al., 2019)
iR-370-3p, miR-26a-5p	Higher expression	Serum of LTBI	(Lyu et al., 2019)

## Discussion

Exosomes are a promising tool for understanding the subtleties of the pathological process of TB, especially when released from cells infected with MTB. As for the structure and composition of exosomes, they have close relations to their function, modulating the host's immune response. Exosomes derived from MTB organelles called MVBs carry lipids, proteins, nucleic acids, and miRNA that are important for MTB to thrive, grow, and suppress the immune response. MTB has been known to use different proteins, such as ESAT-6 and Antigen 85 (Ag85), to control the host's immune response and survive within macrophage cells (Raposo and Stoorvogel, 2013). Some proteins present in MTB exosomes, such as lipoarabinomannan (LAM), may affect apoptosis and cytokine secretion. This interaction may enhance the immunological response to MTB, aligning with the pathogen's survival strategy. Interestingly, this adaptation also serves a critical public health interest (Giri et al., 2010). Therefore, understanding the composition of exosomes is important for understanding how MTB communicates with the host and for developing effective TB diagnosis and treatment strategies (Schorey and Harding, 2016).

As reported in this review, several pieces of evidence have stressed the importance of host miRNAs (Behrouzi et al., 2019), sRNAs (Coskun et al., 2021), and circular RNA (circRNAs) (Wang et al., 2023) in TB. However, more research is needed, particularly in identifying the exosomal miRNAs from MTB. Despite sRNAs having minimal impact on infection and expression rates, MTB specifically produces exosomal miRNAs crucial for modulating the host immune response. These miRNAs can alter several macrophage functions, assisting the pathogen in evading the immune response and perpetuating infection. The influence of exosomal miRNAs on TB's expression and infection process is highly significant and informative for understanding the disease. Shedding light on exosomal miRNAs is crucial as they are potential diagnostic markers and therapeutic targets in TB.

Furthermore, TB diagnosis has improved by considering associated diseases (Condrat et al., 2020). miR-29a is implicated in distinguishing the active form of TB from the latent one, thereby enhancing diagnostic accuracy (Sinigaglia et al., 2020). The potential to apply this approach during therapy is rather significant. Indeed, the possibility of establishing treatment for a critical issue of antibiotic resistance can be evaluated by examining the regulations of different miRNAs. Moreover, miR-125b is crucial for promoting MTB growth by regulating inflammatory signaling and can be safely depleted to boost the host's immunity against the infection (Sun et al., 2021a).

Studies have shown that some miRNAs may be targeted for therapy, as reported in Table 3. Unfortunately, there is scarce available data regarding their involvement in the prognosis of TB. More studies are required to elucidate their significance in diagnostic evaluation and understanding the causative agent of TB (Wang et al., 2023). According to Ma et al. (2020) increasing the concentration of miR- 155 increases the activity of the macrophage-related gene, boosting the ability of macrophages to counteract MTB. miR-125b is widely acknowledged as one of the miRNAs that can suppress inflammation. Thus, miR-125b is widely acknowledged to suppress inflammation; thus, blocking miR-125b will prevent the suppression of the host's immune response. miR-21 plays a vital role in controlling apoptosis and immunity, so suppressing its activity may positively affect immune response capacity and inflammation degree. Modulating specific miRNAs can significantly impact TB progression, with beneficial miRNAs counteracting detrimental ones (Schröder, 2022).

**Table 3:** List of miRNAs as potential biomarkers.

**miRNAs**	**References**
miR-4669-5p	Wang et al., 2023
miR-20b-5p	Zhang et al., 2019
miR-27-3p, let-7a-5p, let-7c-5p, miR-25-3p, miR-98 5p, miR-30a-3p, miR-194-5p, miR-5110	Zhan et al., 2022
miR-17-5p, miR-20b-5p	Tu et al., 2019
miR-1246, miR-2110, miR-28-3p, miR 193b-5p,	Lyu et al., 2019
miR-484, miR-425, miR-96	Alipoor et al., 2019
miR-205-5p, miR-200c-3p, miR-141-3p, miR-483-5p, miR-375	Wang et al., 2017
miR-33a-3p, miR-153-3, miR-373-5p, miR-3120-5p, miR-489-3p, miR-4669-5p	Zhang et al., 2022
miR-143-3p, miR-210-3p, miR 20a-5p and 23b, miR 17, miR 584	Guio et al., 2022

Thus, there is great hope for further developing miRNA investigations related to TB. With the potential for developing miRNA studies in TB, exciting avenues may be explored in the future. Effective, high-throughput miRNA sequencing techniques are anticipated to provide greater insight into miRNA function in TB, alongside advancements in bioinformatics data analysis methods. This progress will likely lead to new technologies in diagnosis and treatment.

## Conclusion

These miRNAs are critically involved in managing the immune system's response to TB, a significant global health concern. These small non-coding RNAs, including miR-155, miR-125b, and miR-29a, play a pivotal role in governing innate and adaptive immunity. They modulate the differentiation of macrophages and T cells and the levels of cytokines. Thus, exosomal miRNAs secreted by MTB are reported to have a crucial function in regulating the host's immune response, allowing the pathogen to avoid elimination by the immune system and persist in the body. Due to the exosome's stability and non-invasive nature, miRNAs have substantial value as biomarkers for distinguishing between active TB and LTBI, contributing to the development of efficient and accurate diagnostic tests. Moreover, given the diverse roles of miRNAs, miRNA-based therapies hold promise for improving immune responses and countering bacterial survival strategies. These approaches offer new ways to combat drug resistance and enhance patient treatment outcomes.

## Author contributions

FM: Conceptualization, Investigation, Writing – original draft, Writing – review & editing. AG: Data curation, Writing – original draft, Writing – review & editing. NB: Formal analysis, Investigation, Writing – review & editing. MF: Formal analysis, Investigation, Writing – review & editing. MD: Data curation, Investigation, Writing – review & editing. MA: Conceptualization, Data curation, Writing – review & editing. MZ: Conceptualization, Formal analysis, Supervision, Writing – review & editing. DN: Supervision, Validation, Writing – review & editing. RDM: Project administration, Resources, Supervision, Validation, Writing – review & editing. GPP: Conceptualization, Validation, Writing – original draft, Writing – review & editing.
